# Valorisation of apple pomace for the development of high-fibre and polyphenol-rich wheat flour cookies

**DOI:** 10.1038/s41598-024-77377-8

**Published:** 2024-10-29

**Authors:** Zahida Naseem, Naseer Ahmad Bhat, Sajad Ahmad Mir

**Affiliations:** 1 Department of Food Science and Technology, University of Kashmir, Srinagar, 190006 India; 2 Design Innovation Centre (DIC), Central University of Kashmir, Ganderbal, 191201 India; 3 Department of Life Science (Microbiology and Food Science and Technology), GITAM (Deemed to be University), Visakhapatnam, 530045 India

**Keywords:** Wheat flour, Apple pomace, Thermal analysis, Color, Rheology, Antioxidant, Health care, Nutrition

## Abstract

Apple pomace, abundant in dietary fibre and polyphenols, often goes unutilized, contributing to environmental pollution as it is discarded in open fields of Jammu and Kashmir. This study aimed to develop functional cookies fortified with apple pomace powder (APP), an industrial by-product. Wheat flour-APP formulations (0%, 5%, 10%, and 15%) were assessed. APP addition notably affected color values and functional properties, enhancing water and oil absorption capacities, swelling power, foam capacity and stability. Phenolic content increased significantly (*p* < 0.05) post-fortification, elevating antioxidant properties. FT-IR spectroscopy identified distinctive chemical components in wheat flour and APP. Sensory evaluation favored cookies with 10% APP, indicating their potential for consumer acceptance. Thus, APP shows promise for producing innovative functional cookies, improving consumer health, utilizing industrial by-products, and reducing waste from apple processing plants, thereby mitigating environmental pollution.

## Introduction

Wheat (*Triticum aestivum* L.) is a staple crop in global temperate regions, serving as a vital source for both human food and livestock feed. Its higher protein content compared to other cereals makes it a primary protein source in human diets^1^. However, during milling, wheat grain loses its bran and germ, which contain numerous health-beneficial components^2^ such as fibre, minerals, vitamin E, vitamin B6, thiamine, folate and phytochemicals, like phenolic compounds^3^. Enhancing the nutritional value of wheat-based products can be achieved by incorporating various bioactive constituents during their preparation. Studies have shown that adding carrot pomace powder, mango peel powder^4^, currant and jostaberry powder^5^ and eggplant to wheat flour can increase its antioxidant activity. Apple pomace, rich in polyphenols and dietary fibre, presents an opportunity to enrich wheat flour-based products such as cookies, cakes and muffins, thereby improving their nutritional and health-promoting properties.

Apples (*Malus domestica*) are widely favored fruits cultivated across temperate regions worldwide. Global apple production totals approximately 83.14 million tonnes from nearly 4.93 million hectares of land^6^. India ranks as the fifth-largest apple producer globally, contributing around 2.72% of the total global apple production^7^. Apple cultivation is prominent in Indian states like Jammu and Kashmir, Uttaranchal and Himachal Pradesh. During apple processing, approximately two-thirds of the apple’s fresh weight is removed as juice, leaving behind pomace as solid waste. Apple pomace is rich in dietary fibre and polyphenols, exhibiting high antioxidant activity due to phytochemicals like flavonoids, phenolics and flavan-3-ols^8^. These polyphenols possess potent antioxidant, anticancer, antimicrobial and cardiovascular-protective properties. However, the major apple processing plants in Jammu and Kashmir generate significant quantities of unused apple pomace, contributing to environmental pollution.

In light of these considerations, the present study aims to develop functional cookies using wheat flour and apple pomace, while also conducting physicochemical, functional, bioactive and sensory characterizations of the resulting product. The incorporation of apple pomace into wheat flour for the production of functional cookies is expected to enhance techno-functional attributes and enrich bioactive compounds in the final product. Specifically, it is anticipated that the addition of apple pomace will augment the antioxidant activity, dietary fibre content and polyphenolic composition of the cookies. Additionally, the utilization of apple pomace in cookie production will offer a sustainable solution for reducing environmental pollution associated with apple processing waste.

## Materials and methods

### Sample collection

**Fig. 1:**
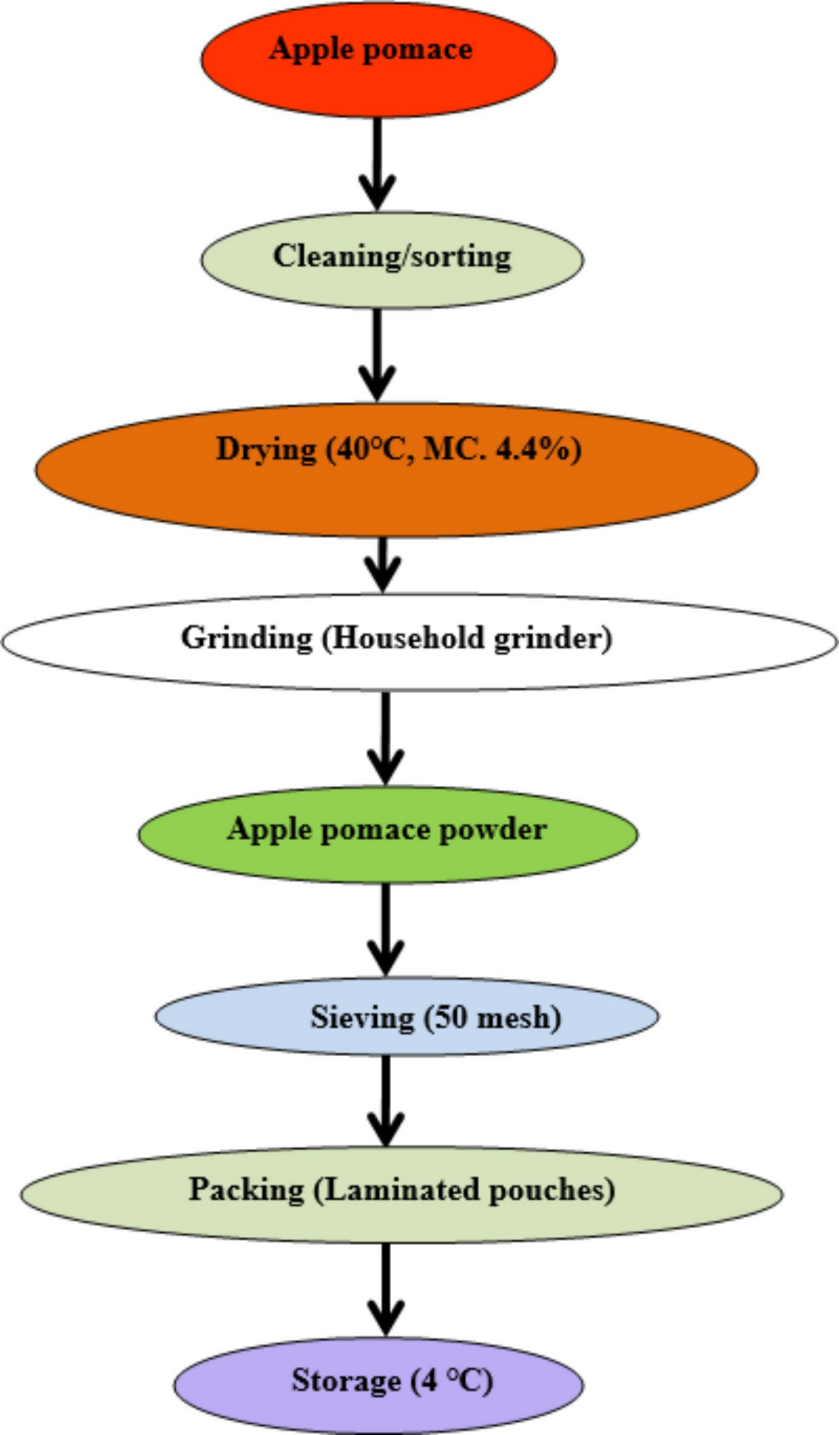
Flow chart showing preparation of apple pomace powder.

A soft wheat cultivar (SKW-355) was sourced from Sher-e-Kashmir University of Agricultural Sciences and Technology, Kashmir (SKUAST-K), India. The Golden delicious apples were purchased from a local marketplace and transported to the pilot processing plant of the Department of Food Science and Technology, University of Kashmir, India. Apples were carefully selected, with bruised and blemished ones discarded. After thorough washing, coring was performed using a clean, sharp knife. The apples were then sliced and crushed to extract pulp, which was subsequently pressed to extract juice. The resulting apple pomace was dehydrated in a cabinet dryer at 40 °C until its moisture content reached 4.4%. Once dried, the apple pomace was ground into a fine powder using a household grinder and sieved through a 50-mesh screen to achieve a reduced particle size. The apple pomace powder (APP) was then stored in airtight polyethylene bags at refrigerated temperatures (4 °C) for further analysis. A flowchart detailing the apple pomace production process is provided in (Fig. 1).

### Preparation of formulations

Formulations of wheat flour substituted with APP were prepared. The wheat flour was replaced by different levels of apple pomace. Wheat flour without addition of APP was kept as control and labeled as WF (0%) while as, wheat flour replaced with 5%, 10% and 15% apple pomace powder were labeled as WFP_1_, WFP_2_ and WFP_3_ respectively.

### Preparation of cookies

To prepare the cookies, a mixing bowl was used to combine 100 g of shortening with 250 g of wheat flour or wheat flour mixed with varying concentrations of apple pomace powder. This mixture was thoroughly blended in a laboratory mixer (Model GF-101, Taichung City 421, Taiwan) for 2 min. Subsequently, sugar (112.5 g), skim milk powder (30 g), salt (1.5 g), baking powder (1.5 g), and liquid egg white (25 g) were added and mixed again until thoroughly incorporated. Water was then added to achieve the desired dough consistency. The dough was allowed to rest for 10–15 min before being rolled out to a uniform thickness of 6 mm using a sheeter (Model JDR 520, Dolar Engineering Industries PVT. LTD, Bangalore, India). Round cookies were cut from the dough sheet using a cutter with a 5.5 cm diameter. These cookies were baked in a hot air oven (Dolar Engineering Industries PVT. LTD, Bangalore, India) at approximately 150–160 °C for 10 min and then cooled to room temperature. The baked cookies were carefully packed in airtight laminated pouches and labeled as follows: C_0_ (wheat flour cookies without apple pomace), C_1_ (wheat flour cookies with 5% apple pomace), C_2_ (wheat flour cookies with 10% apple pomace) and C_3_ (wheat flour cookies with 15% apple pomace). The cookies were stored at ambient temperature (22–25 °C) until further analysis.

### Proximate composition of raw material and cookies

The proximate composition (protein, crude fibre, moisture content, fat and ash) of the samples was determined according to^9^ standard methods. Each analysis was run in triplicate (*n* = 3).

### Physicochemical properties of raw material

#### Water and oil absorption capacity

The protocol outlined by^10^ was employed for assessing the oil and water absorption capacities of wheat flour, apple pomace and various formulations. Water absorption capacity (WAC) and oil absorption capacity (OAC) were quantified as the percentage of water or oil absorbed per gram of sample, respectively. Measurements were performed in triplicates (*n* = 3).

$$WAC~or~OAC~\% =\frac{{\left( {Weight~of~residue~\left( g \right) - weight~ofsample~~\left( g \right)} \right)}}{{weight~ofsample~~\left( g \right)}} \times 100$$

#### Swelling power

Swelling power (SP) was determined by using the method described by^11^ with minor modification. Measurements were carried out in triplicates (*n* = 3). The swelling power was determined as:

$$SP=\frac{{weight~of~the~paste~~\left( g \right)}}{{weight~of~dry~flour~~\left( g \right)}}$$

#### Foam stability (FS) and foaming capacity (FC)

Foaming capacity and foam stability was determined by the method described by^12^. The FC was expressed as a percentage rise in volume:

$$FC=\frac{{\left( {Volume~after~whipping~\left( {mL} \right) - Volume~before~whipping~\left( {mL} \right)} \right)}}{{Volume~before~whipping~\left( {mL} \right)}} \times 100$$

To determine the foam Stability (FS) the foam volume was noted 1 h after whipping. It is expressed as a percentage of the initial foam volume.

$$FS=\frac{{Foam~volume~after~standing~time~\left( {mL} \right)}}{{Initial~foam~volume~\left( {mL} \right)}} \times 100$$

### Fourier transform infrared (FTIR) spectroscopy

The samples underwent structural characterization utilizing FTIR-ATR (Fourier Transform Infrared Attenuated Total Reflectance) spectroscopy, conducted with a Cary 630 FTIR instrument from Agilent Technologies, Stevens Creek Blvd. Santa Clara, USA. The analysis was carried out at a temperature of 25b °C with a resolution of 4 cm using Resolution Pro software version 2.5.5 (Agilent Technologies, Stevens Creek Blvd. Santa Clara, USA).

### Total flavonoid content of raw material

The flavonoid content (TFC) of the raw material was assessed following the methodology outlined by^13^. A standard curve of catechin was established and the flavonoid content was quantified and reported in milligrams of catechin equivalents per gram (mg CE/g). Three readings (*n* = 3) were made for each sample.

### Total phenolic content of raw material and cookies

The total phenolic content (TPC) of the samples was determined following the modified procedure outlined by^14^. A standard curve of gallic acid was employed to quantify the TPC of the samples, expressed in milligrams of gallic acid equivalents (GAE) per gram of sample. Three readings (*n* = 3) were made for each sample.

### Antioxidant properties of raw material and cookies

#### DPPH (2,2-diphenyl-2-picrylhydrazyl) radical scavenging activity

DPPH radical scavenging activity of the samples was determined by the modified procedure of^15^. Absorbance was measured at wavelength of 517 nm using spectrophotometer after 30 min of incubation. Readings were taken in triplicates for each sample (*n* = 3). Antioxidant activity was calculated as % inhibition of DPPH radical by using the following formula:

$${\text{DPPH~radical~scavenging~activity~}}\left( {\text{\% }} \right)=\frac{{[1 - \left( {{\text{Absorbance~of~sample}}} \right]}}{{\left[ {{\text{Absorbance~of~control}}} \right]}} \times 100$$

#### Reducing power

Reducing power of methanol extracts of samples was assessed using the method described by^16^. The absorbance was observed at 700 nm against methanol with ascorbic acid taken as a standard. A higher reducing power is indicated by higher absorbance. Measurements were performed in triplicates (*n* = 3).

#### Inhibition of lipid peroxidation

The method described by^17^ was utilized to assess lipid peroxidation in the samples. Lipid peroxidation was quantified as the percentage of inhibition and calculated by using the following formula.

$${\text{\% ~Inhibition}}=\frac{{[1 - \left( {{\text{A~of~sample~t}}=30} \right]}}{{\left[ {{\text{A~of~control}}} \right]}} \times 100$$

#### Metal chelating (Fe2+) activity

Metal chelating activity of samples was calculated as per the method described by^18^. The chelating activity of the sample extract for Fe^2+^ was measured as follows:

$${\text{Iron~}}\left( {{\text{Fe}}2+} \right){\text{~chelating~activity~\% }}=\frac{{[1 - \left( {{\text{As~}}562} \right]}}{{\left[ {{\text{Ac}}562} \right]}} \times 100$$

Where, A_S562_ and A_C562_ is absorbance of sample and control measured at 562 nm respectively. Measurements were performed in triplicates (*n* = 3).

### Pasting properties

The pasting properties of wheat flour and formulations were analyzed using a Rapid Visco Analyzer (Tech Master, Perten Instruments Pvt Ltd, Macquarie Park, Australia). A mixture of 3 g of flour sample and 25 g of distilled water was prepared in the RVA sample canister, resulting in a total suspension of 28 g. The flour suspensions were initially held at 50 °C for 1 min. Subsequently, they were heated to 95 °C and maintained at this temperature for 3 min before being cooled back to 50 °C over a period of 4 min. The suspensions were then held at 50 °C for an additional 2 min. Various RVA parameters, including peak viscosity, pasting temperature, trough viscosity (minimum viscosity at 95 °C), breakdown viscosity, final viscosity (viscosity at 50 °C), and setback viscosity were recorded. The results were reported in centipoise (cP).

### Differential scanning calorimetry (DSC)

The thermal characteristics of wheat flour and formulations were assessed using a DSC822 instrument (Mettler-Toledo, Neuchatel, Switzerland). Each sample, consisting of 5 mg (on a dry basis), was placed into a platinum pan with a 40 µL capacity. The pan was then hermetically sealed and an equilibration period of 1 h was allowed. Subsequently, the samples were subjected to heating at a rate of 10 °C/min over a temperature range of 25 to 200 °C. Transition temperatures, including onset (To), peak (Tp) and conclusion (Tc) temperatures, as well as the enthalpy of gelatinization (ΔH), were determined based on the dry weight of the wheat flour. Measurements were performed in triplicate (*n* = 3) and the average values were reported.

### Rheology

The rheological properties of wheat flour and formulations were evaluated using a dynamic rheometer (MCR102, Anton Paar, Graz, Austria). Each dough sample (2 gm) was allowed to rest for 15 min in a polyethylene bag before analysis to assess variations in storage modulus (G′) and loss modulus (G″). A frequency sweep ranging from 0.1 to 100 Hz was conducted at a constant strain within the linear viscoelastic range (γ = 1 Pa) at a temperature of 25 °C. Measurements were performed in triplicate (*n* = 3) and the average values were reported.

### Physical characteristics of cookies

The physical characteristic (thickness, weight, diameter and spread ratio) of cookies was measured as per the procedure described by^19^. Readings were taken in triplicates (*n* = 3) and the average values were reported.

#### Texture

TA-XT Plus, texture analyzer (Stable Micro Systems, Vienna Court, UK) was used for determining the hardness of cookies. The parameters that were set for texture measurement include probe pre-test speed = 1 mm/s, test speed = 3 mm/s, post-test speed = 10 mm/s and trigger force = auto. Each cookie sample was centrally placed on the base plate and the blade travel distance was kept as 10 mm. The peak force used to break the cookies represented the hardness. The average values of three readings were reported (*n* = 3).

#### Color

The color (L*, a*, b*) value of APP, WF, formulation and cookies were measured by a colorimeter (12 MM Aperture U 59, New York, USA). Three replicates (*n* = 3) were taken for each sample. Color differences (ΔE*) between samples was calculated according to the CIE formula.

$$\Delta E* = \left[ {(\Delta L*)^{2} + {\text{ }}(\Delta a*)^{2} + {\text{ }}(\Delta b*)^{2} } \right]^{\raise.5ex\hbox{$\scriptstyle 1$}\kern-.1em/ \kern-.15em\lower.25ex\hbox{$\scriptstyle 2$} }$$

### Sensory evaluation

The sensory assessment of cookies involved a panel of 25 semi trained evaluators selected from the Department of Food Science and Technology at the University of Kashmir, India. Each evaluator was presented with four samples, each identified by a unique code. Evaluators assessed various sensory attributes including texture, appearance, flavor, mouthfeel, and overall acceptability using a 9-point hedonic scale.

### Statistical analysis

The means and standard deviations of all triplicate (*n* = 3) measurements were calculated for each analysis in the current study. One-way analysis of variance (ANOVA) was conducted to determine significant differences among the mean values. Subsequently, Duncan’s LSD test was employed to identify specific differences, utilizing the commercial statistical package SPSS ver. 11.5 (SPSS Inc., Chicago, IL, USA), with a significance level set at 5% (*p* < 0.05).

## Results and discussion

### Proximate composition of WF, APP and formulations

Table 1 presents the proximate composition of WF, APP, and the formulations. Moisture content was observed as 4.4% for APP, and ranged from 12.4 to 13.6% for WF with 0% APP (WF), WF containing 5% APP (WFP1), WF with 10% APP (WFP2), and WF with 15% APP (WFP3), respectively. Ash content of WF, APP, and the formulations fell within the range of 0.40–1.50%, with the highest found in APP and the lowest in WF. Kohajdova et al.^20^ reported ash content for apple fibre powder as 2.07% and for wheat flour as 0.46%, aligning with our study results.

**Table 1:** Proximate composition and functional properties of, wheat flour, APP and formulations (*n* = 3).

	APP	WF	WFP_1_	WFP_2_	WFP_3_
Moisture (%)	4.40 ± 0.41^a^	13.6 ± 0.33^d^	12.8 ± 0.24^c^	12.4 ± 0.16^bc^	12.0 ± 0.08^b^
Ash (%)	1.50 ± 0.49^b^	0.53 ± 0.37^a^	0.53 ± 0.29^a^	0.50 ± 0.24^a^	0.50 ± 0.01^a^
Protein (%)	1.43 ± 0.04^a^	9.62 ± 0.03^e^	9.21 ± 0.02^d^	8.80 ± 0.01^c^	8.39 ± 0.01^b^
Fat (%)	1.80 ± 0.08^b^	1.56 ± 0.02^a^	1.59 ± 0.02^a^	1.58 ± 0.04^a^	1.57 ± 0.03^a^
Fibre (%)	31.25 ± 0.01^e^	0.66 ± 0.02^a^	2.19 ± 0.03^b^	3.71 ± 0.03^c^	5.26 ± 0.05^d^
TFC mg CE/g	2.82 ± 0.21^e^	0.26 ± 0.10^a^	0.37 ± 0.04^b^	0.58 ± 0.12^c^	0.75 ± 0.03^d^
WAC (g/g)	3.35 ± 0.27^d^	1.01 ± 0.05^a^	1.15 ± 0.02^a^	1.23 ± 0.10^b^	1.40 ± 2.0^c^
OAC (g/g)	1.05 ± 0.18^a^	1.03 ± 0.13^a^	0.97 ± 0.10^a^	0.95 ± 0.10^a^	0.92 ± 0.82^a^
SP (g/g)	4.66 ± 0.08^bc^	4.31 ± 0.13^a^	4.44 ± 0.14^ab^	4.63 ± 0.15^abc^	4.83 ± 0.17^c^
FC (%)	24.0 ± 0.82^a^	46.0 ± 1.6^d^	40.0 ± 2.0^c^	38.0 ± 0.8^c^	28.0 ± 0.16^b^
FS (%)	25.0 ± 1.6^a^	48.0 ± 1.2^c^	65.0 ± 2.4^d^	26.0 ± 0.81^a^	42.0 ± 0.82^b^
Color					
L*	61.16 ± 0.28^a^	85.84 ± 0.44^e^	81.27 ± 0.41^d^	78.01 ± 0.50^c^	75.70 ± 0.42^b^
a*	3.82 ± 0.20^e^	– 0.86 ± 0.07^a^	0.58 ± 0.06^b^	1.20 ± 0.09^c^	1.82 ± 0.07^d^
b*	29.55 ± 1.31^c^	18.74 ± 0.23^a^	18.87 ± 0.60^a^	18.97 ± 0.34^a^	19.87 ± 0.46^b^
ΔE	–	27.30 ± 0.09^d^	22.99 ± 0.08^c^	20.06 ± 0.06^b^	17.58 ± 0.06^a^

Results are expressed as means (*n* = 3) ± standard deviation.

*WAC* water absorption capacity; *OAC* oil absorption capacity; *SP* swelling power; *FC* foaming capacity; *FS* foam stability; *APP* apple pomace powder; *WF*,* WFP*_*1*_, *WFP*_*2*_* and WFP*_*3*_ wheat flour sample containing 0%, 5%, 10% and 15% APP respectively; *C*_*0*_, *C*_*1*_, *C*_*2*_* and C3* cookies containing 0%, 5%, 10% and 15% APP, respectively.

Values followed by same letter in a row do not differ significantly (*p* < 0.05).

The protein content of WF (9.62%) was significantly (*p* < 0.05) higher than that of APP (1.43%), resulting in a notable (*p* < 0.05) decrease in protein content in formulations with increasing concentrations of APP. Ahmad et al.^21^ also reported a similar decrease in protein content after adding carrot pomace to wheat flour. Crude fibre and fat content of APP were recorded as 31.23% and 1.80%, respectively, while for WF, these values were 0.65% and 1.50%, respectively. Yadav & Gupta^22^ reported the fibre content of APP as 30.86%, that is in line with our study. The crude fibre content significantly (*p* < 0.05) increased with the rise in APP concentration in WF, likely due to the higher fibre content in APP compared to wheat flour.

### Color of WF, APP and formulations

Table 1 provides the Hunter color in terms of L* (lightness), a* (redness), and b* (yellowness) values of WF, APP and the formulations. The L* value of the flour blends exhibited a significant decrease (*p* < 0.05) from 85.84 to 75.70 as the level of APP increased from 0 to 15%. In contrast, the a* value of the flour blends showed a significant increase (*p* < 0.05) from − 0.86 to 1.82, while the b* value exhibited a non-significant increase (*p* > 0.05) from 18.74 to 19.87 with the addition of APP. The decrease in L* value and the increase in a* and b* values of the formulations may be attributed to the brown color of apple pomace powder. This suggests that as the concentration of apple pomace increased in the formulations, the resulting products exhibited a darker color (lower L* value), with increased redness (higher a* value) and a slight increase in yellowness (higher b* value).

The total color difference (Δ*E*) varied significantly among the samples from 17.58 to 27.30. The highest Δ*E* was found in WFP_3_ and lowest in case of WF. The more the value of Δ*E* the more is the difference in total color with respect to control (APP) and vice versa.

This can be attributed to due to darker color of the apple pomace that may have generated due to the Maillard during the dying of apple pomace^23^.

### Physicochemical properties of WF, APP and formulations

#### Water and oil absorption capacity

Table 1 displays the values of oil and water absorption capacity of APP, WF and the formulations. The water absorption capacity (WAC) of APP (3.35 g/g) was significantly (*p* < 0.05) higher than that of WF (1.01 g/g). This difference in WAC could be attributed to the abundance of hydroxyl groups present in the fibre structure of APP, facilitating increased water interaction through hydrogen bonding^24^. The WAC significantly (*p* < 0.05) increased from 1.01 to 1.40 g/g with the addition of APP to WF from 0 to 15%. The highest value was observed in the formulation containing 15% APP while lowest value was found in wheat flour. Similar trend was found by^25^ in their study, while evaluating the nutritional and quality characteristics of black carrot fortified instant noodles. The elevated WAC observed in the formulations could potentially benefit bakery products by reducing moisture loss and thus staling.

The oil absorption capacity (OAC) helps in the improvement of mouth feel and the retention of flavor. The oil absorption capacity (OAC) of APP (1.05 g/g) did not significantly differ (*p* > 0.05) from that of WF (1.03 g/g), and it was observed to decrease non-significantly (*p* > 0.05) from 0.97 to 0.92 g/g after the incorporation of APP. The decreased OAC of APP and the formulations compared to WF may be attributed to the higher protein content in WF, which serves as a hydrophobic material with more available hydrophobic sites for oil binding^26^.

#### Swelling power

Table 1 presents the swelling power (SP) of the samples. The SP of APP (4.66%) was significantly (*p* < 0.05) greater than that of WF (4.31%), indicating the high water absorption capacity of APP during heating. This finding aligns with similar results reported by^19^ for cookies from composite wheat–sesame peels flours. Incorporating APP into wheat flour from 10 to 15% resulted in a significant (*p* < 0.05) increase in the SP of wheat flour from 4.31 to 4.83%. The high SP of WF containing APP can be attributed to APP’s richness in fibre. Ahmad et al.^21^ also reported that the addition of carrot pomace powder to wheat flour increased its swelling capacity, consistent with the findings of our study.

#### Foaming capacity and foam stability

Foaming capacity (FC) and foam stability (FS) of WF, APP and formulations is presented in (Table 1). The FC and FS of WF (46% and 65%, respectively) were significantly (*p* < 0.05) higher than those of APP (24% and 25%, respectively). Consequently, the addition of APP to WF significantly (*p* < 0.05) reduced the FC and FS of the formulations. FC primarily relies on proteins, which create a continuous thin film around gas bubbles within the foam. Proteins also play a crucial role in stabilizing air bubbles by lowering the surface tension at the air-water interface. The decrease in FC and FS may be attributed to the dilution of protein content in wheat flour with the increasing incorporation of APP. Ahmad et al.^21^ similarly noted a decline in the foaming capacity of wheat flour following the addition of carrot pomace powder. Further the presence of stabilizing agent like pectin, in food foams and emulsions may influences the mechanical resistance of the interface, leading to improved stability against rupture^27^.

### Fourier transform infrared spectroscopy (FT-IR) WF, APP and formulations

**Fig. 2:**
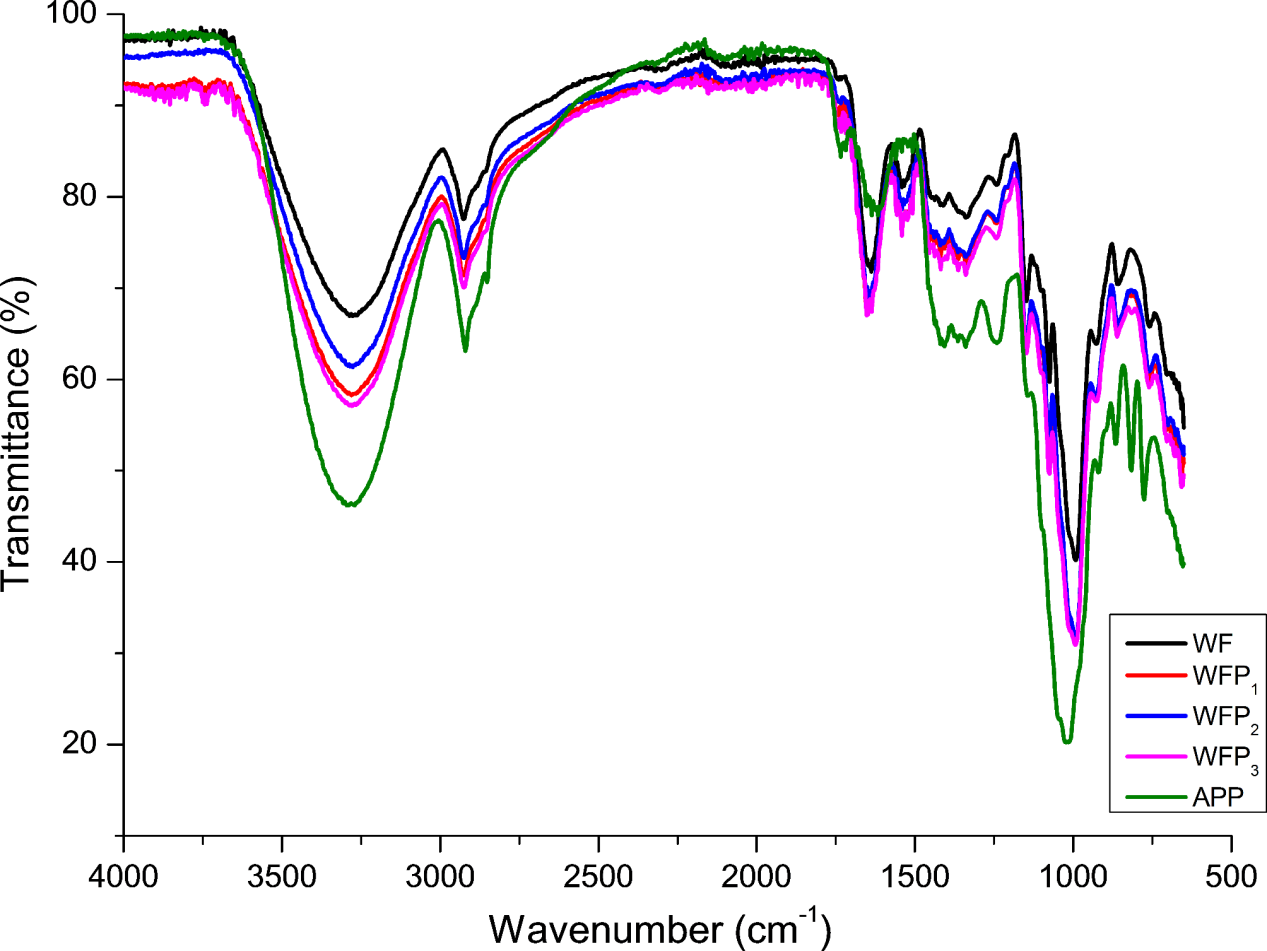
FT-IR spectra of wheat flour, apple pomace and wheat flour-apple pomace formulations. *APP* apple pomace powder; *WF*,* WFP*_*1*_, *WFP*_*2*_* and WFP*_*3*_ wheat flour sample containing 0%, 5%, 10% and 15% apple pomace powder, respectively.

The FT-IR spectra of WF, APP and formulations are shown in (Fig. 2). The major spectral bands observed in wheat flour between 3000 and 3600 cm^− 1^, 2800–3000 cm^− 1^, 950–1030 cm^− 1^ and 1600–1800 cm^− 1^, were allotted to O–H stretch, C–H stretch, C–O–C stretch and the bending mode of water, respectively^2^. In case of apple pomace, the main spectral bands between 1630 –1600 cm^− 1^, 1300–1450 cm^− 1^, 1236–1268 cm^− 1^, 1000–1030 cm^− 1^, were respectively assigned to COO^−^ asymmetric stretching (pectin ester group), CH deformation from ring vibration (polysaccharides, pectin, cellulose) C–O stretching (pectin) and C–O stretching and C–C stretching (pectin, cellulose). The cell walls of fruits are composed of different polysaccharides including pectin, cellulose and hemicelluloses and smaller quantities of phenolic esters, glycoproteins, minerals, enzymes etc. From the graph (Fig. 2) it is clear that the intensities of all the spectral bands were found to surge with the rise in the concentration of APP in WF. Some shifts in the peak position between wheat flour and apple pomace were also observed which might be due to the different concentration, type and structure of components present in the APP. The possible reason for the variation in peak intensities between WF, APP and wheat flour-apple pomace blends could be attributed to the higher amount of polysaccharides such as dietary fibre and cellulose in apple pomace as compared to wheat flour. Further the difference in the peaks may be due to the presence of bioactive compounds like chlorogenic acid, phloridzin, hyperoside, rutin, avicularin, quercetin, isoquercitrin, and reynoutrin in apple pomace. Similar results were reported by^28^ on cookies enriched with *Tinospora cordifolia* stem powder.

### Pasting properties of WF and formulations

Table 2 illustrates the pasting properties of WF and the formulations. The analyzed pasting properties include peak viscosity (PV), trough viscosity (TV), breakdown viscosity (BDV), setback viscosity (SBV), final viscosity (FV), and pasting temperature (PT). PV and TV were observed to significantly increase (*p* < 0.05) after the incorporation of APP (0–10%) compared to WF. However, with a higher level of APP incorporation (15%), PV decreased significantly (*p* < 0.05). As evident from the data (Table 2), BDV, SBV and FV were also observed to decrease significantly (*p* < 0.05) with the increasing level of APP addition to WF. This decline in pasting properties was attributed to fewer available starch granules for gelatinization after the incorporation of APP. Additionally, APP, being a rich source of dietary fibre, holds more water than wheat flour, potentially reducing the amount of water available for starch granules to fully gelatinize^29^. The highest BDV value was observed in wheat flour (952 cP), as the incorporation of dietary fibre decreased starch swelling, thereby increasing paste resistance. The lower SBV value reflects a lesser degree of retrogradation in a starch paste. Blanco et al.^30^ studied the incorporation of dietary fibre in cookie dough and reported similar results for pasting properties. The pasting temperature of formulations decreased significantly (*p* < 0.05) with the increase in the addition of APP. This decrease in pasting temperature may be attributed to the varying gelation temperature of the fibre fractions.

**Table 2:** Pasting and thermal properties wheat flour and formulations (*n* = 3).

	WF	WFP_1_	WFP_2_	WFP_3_
Peak viscosity (cP)	1859 ± 81.65^ab^	2015 ± 82.06^c^	1986 ± 8.16^bc^	1762 ± 1.63^a^
Trough viscosity (cP)	907 ± 82.47^a^	1327 ± 81.24^c^	1464 ± 7.76^d^	1113 ± 2.45^b^
Breakdown viscosity(cP)	952 ± 82.06^c^	688 ± 80.43^b^	522 ± 8.58^a^	449 ± 0.82^a^
Final viscosity (cP)	2544 ± 82.87^b^	2498 ± 81.24^b^	2489 ± 4.50^b^	2312 ± 2.05^a^
Setback viscosity (cP)	1637 ± 163^c^	1171 ± 81.65^b^	1025 ± 16.33^a^	1001 ± 2.05^a^
Pasting temp (°C)	84.3 ± 0.82^bc^	70.25 ± 0.20^a^	70.9 ± 0.16^a^	85.50 ± 0.08^c^
Onset (°C)	76.44 ± 8.2^a^	83.33 ± 0.82^ab^	90.40 ± 8.12^ab^	94.27 ± 8.25^b^
Peak (°C)	107.20 ± 1.6^a^	107.53 ± 0.77^a^	110.98 ± 8.16^a^	112.33 ± 0.06^a^
End set (°C)	127.60 ± 0.08^a^	125.81 ± 0.01^a^	154.50 ± 0.04^b^	160.60 ± 0.02^c^
Enthalpy (J/g)	6.00 ± 0.02^c^	5.01 ± 0.03^b^	4.75 ± 0.05^ab^	4.21 ± 0.12^a^

Results are expressed as means (*n* = 3) ± standard deviation.

*APP* apple pomace powder; *WF*,* WFP*_*1*_, *WFP*_*2*_* and WFP*_*3*_ wheat flour sample containing 0%, 5%, 10% and 15% APP respectively; *C*_*0*_, *C*_*1*_, *C*_*2*_* and C3* cookies containing 0%, 5%, 10% and 15% APP, respectively.

Values followed by same letter in a row do not differ significantly (*p* < 0.05).

### Total flavonoids content

The total flavonoid content (TFC) of APP, WF and formulations is presented in Table 1. The TFC of AP and WF was observed as 4.82 and 0.26 mg CE/g, respectively. The addition of APP increased the TFC of the formulations from 0.37 to 0.75 CE/g. Zhang et al.^31^ have reported similar results for the flavonoid content of apple pomace. The flavonoid content of wheat flour has been reported as 0.07 mg CE/g which is lower than that observed in the present study. The difference was attributed to the difference in the wheat varieties and to the fact that the refined wheat flour was used in this study.

### Differential scanning calorimetry (DSC)

The thermal transition temperatures and gelatinization enthalpies of WF and formulations are presented in Table 2. The onset temperature (To), peak temperature (Tp), endset temperature (Te) and enthalpy change (ΔH) of the samples increased within the ranges of 76.44–94.27 °C, 107.53–112.33 °C, 127.60–160.60 °C, and 4.21–6.00 J/g, respectively. The incorporation of APP into WF led to an elevation in the thermal transition temperatures across all samples. However, the gelatinization enthalpies of the formulations experienced a significant reduction (*p* < 0.05) compared to WF. The high water-binding capacity of dietary fibre competes for water with starch, thereby limiting the availability of water for complete swelling of starch granules. This phenomenon may account for the increased gelatinization temperatures observed in wheat flour containing APP. Additionally, the interaction between starch and non-starch polysaccharides may enhance water retention, promoting greater mobility during heating and subsequently lowering gelatinization enthalpies^32^. Furthermore, the decrease in gelatinization enthalpy could be attributed to the synergistic interaction between starch and fibre, leading to the formation of a more stable structure^33^.

### Rheology

**Fig. 3:**
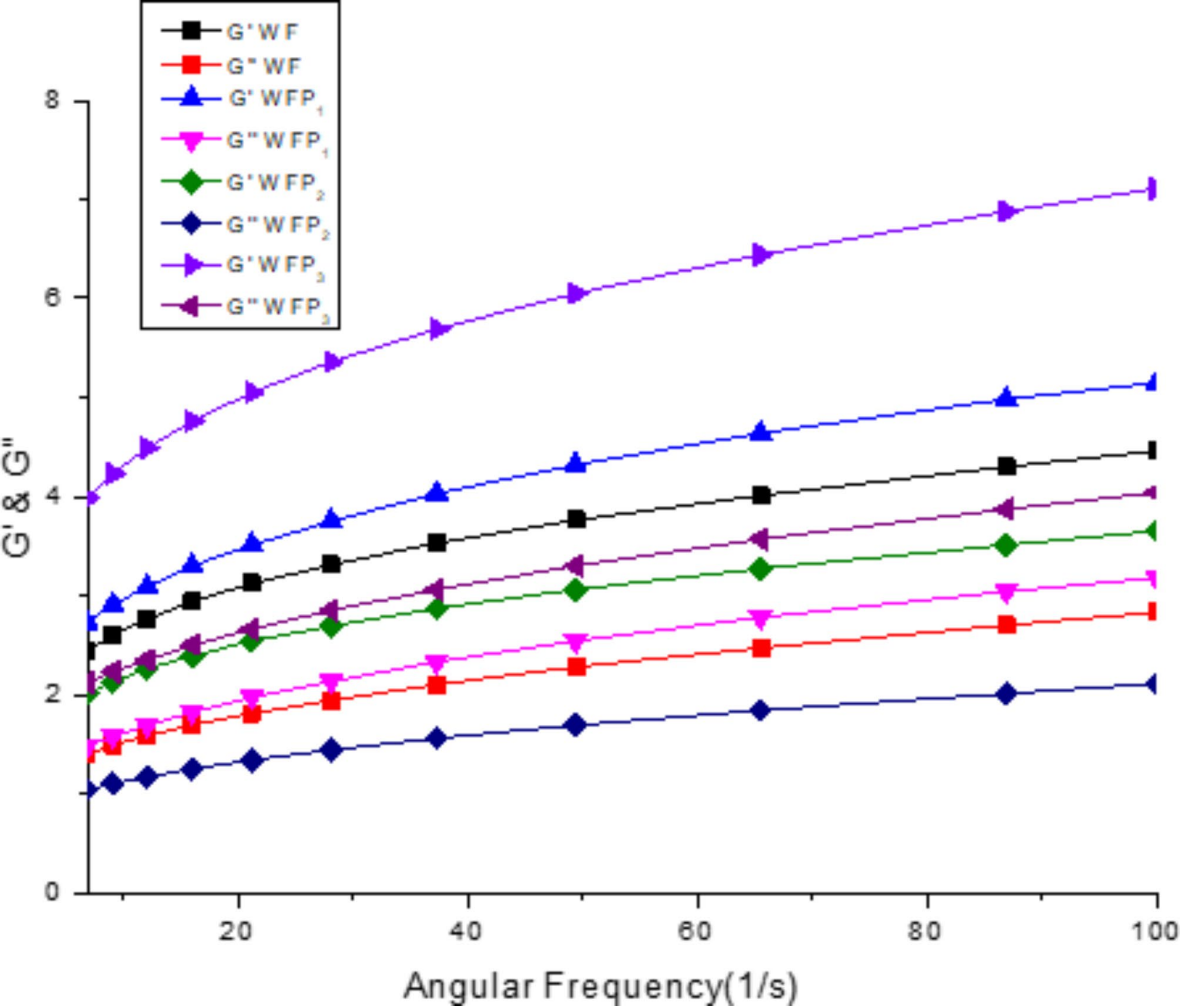
Rheology of wheat flour and formulations. *WF*,* WFP*_*1*_, *WFP*_*2*_* and WFP*_*3*_ wheat flour sample containing 0%, 5%, 10% and 15% apple pomace powder, respectively.

Figure 3 illustrates the dynamic moduli, namely storage modulus (G′) and loss modulus (G″), of WF and the formulations. The storage and loss moduli exhibited enhancement with increasing frequency from 0 to 100/s, indicating frequency-dependent behaviour. This phenomenon could be attributed to the relatively high overall chain mobility within the network of wheat flour doug^34^. Moreover, throughout the frequency sweep, the storage modulus of all samples predominated over the loss modulus, indicating that the dough samples possessed more solid or elastic characteristics. The behaviour of dough samples containing APP slightly differed from the control dough, exhibiting a slightly stiffer nature with increased APP content. This impact of APP on the rheological properties of dough aligns with previous research studies involving the incorporation of carrot pomace^21^ and water-insoluble date fibre into wheat flour dough.

### Total phenolic content of WF, APP, formulations and cookies

Table 3 presents the total phenolic content (TPC) of APP, WF, formulations and cookies. The TPC of APP (1.04 mg GAE/g) was significantly (*p* < 0.05) higher than that of WF (0.28 mg GAE/g). This difference in TPC may be attributed to phenolic acids, which are one of the primary groups of phenolic compounds found in fruits, likely contributing to the higher TPC observed in APP^35^. Additionally, the absence of bran in refined wheat flour, which is a concentrated source of polyphenols, may contribute to the lower phenolic content in WF compared to APP.

**Table 3:** TPC and antioxidant properties of wheat flour, APP, formulations and cookies (*n* = 3).

	TPC (mg GAE/g)	DPPH (% inhibition)	Reducing power (mg AAE/g)	Lipid peroxidation (% inhibition)	Metal chelating activity (%)
APP	1.04 ± 0.82^g^	83.38 ± 0.08^h^	0.66 ± 0.08^d^	67.0 ± 2.04^e^	1.3 ± 0.73^a^
WF	0.28 ± 0.07^a^	48.54 ± 0.07^a^	0.17 ± 0.03^a^	54.0 ± 0.82^b^	1.1 ± 0.65^a^
WFP_1_	0.47 ± 0.08^c^	52.18 ± 0.03^b^	0.20 ± 0.04^a^	59.4 ± 0.08^cd^	1.5 ± 0.57^ab^
WFP_2_	0.70 ± 0.04^d^	54.51 ± 0.02^c^	0.39 ± 0.02^b^	61.7 ± 0.22^d^	1.7 ± 0 .49^b^
WFP_3_	0.86 ± 0.01^e^	59.32 ± 0.01^d^	0.44 ± 0.01^bc^	65.1 ± 0.31^e^	1.9 ± 0.41^b^
C_0_	0.40 ± 0.00^b^	58.45 ± 0.82^d^	0.41 ± 0.02^b^	50.0 ± 0.31^a^	3.5 ± 0.82^c^
C_1_	0.65 ± 0.01^d^	62.68 ± 1.2^e^	0.45 ± 0.03^bc^	53.0 ± 0.33^ab^	3.7 ± 0.08^c^
C_2_	0.88 ± 0.02^ef^	65.45 ± 0.04^f^	0.48 ± 0.04^bc^	55.0 ± 0.40^bc^	4.0 ± 0.24^cd^
C_3_	0.94 ± 0.03^f^	70.69 ± 0.01^g^	0.51 ± 0.05^c^	57.0 ± 0.34^c^	4.2 ± 0.16^d^

Results are expressed as means (*n* = 3) ± standard deviation.

*TPC* total phenolic content; *APP* apple pomace powder; *WF*,* WFP*_*1*_, *WFP*_*2*_* and WFP*_3_ wheat flour sample containing 0%, 5%, 10% and 15% APP respectively; *C*_*0*_, *C*_*1*_, *C*_*2*_* and C3* cookies containing 0%, 5%, 10% and 15% APP, respectively.

Values followed by same letter in a column do not differ significantly (*p* < 0.05).

The TPC of formulations increased significantly (*p* < 0.05) from 0.28 to 0.86 mg GAE/g with the incorporation of APP. This increase could be linked to the release of components from apple pomace, which is rich in polyphenols.

Furthermore, the TPC content of cookies significantly increased (*p* < 0.05) upon baking. At higher temperatures, the formation of melanoidins during the Maillard reaction contributed to the elevation of TPC in cookies^36^.

### Antioxidant properties of WF, APP, formulations and cookies

#### DPPH (2, 2-diphenyl-2- picrylhydrazyl) scavenging activity

DPPH scavenging activity of raw ingredients and cookies is given in Table 3. The results indicate that an increase in the quantity of APP in both WF and cookies results in a corresponding increase in the percent inhibition of DPPH. APP exhibited greater DPPH radical scavenging activity (83.38%) and consequently, increasing its fraction from 0 to 15% in WF significantly (*p* < 0.05) elevated the DPPH radical scavenging activity from 48.54 to 59.32%. This finding is consistent with the results reported by^22^, who noted the DPPH radical scavenging activity of APP as 82.74%, aligning closely with our study.

Baking significantly (*p* < 0.05) increased the DPPH radical scavenging activity of all the cookies. This phenomenon can be attributed to the generation of Maillard reaction products during baking. This reaction induces the formation of compounds and reductone (enaminone) structures with antioxidant activity, such as reactive oxygen species scavenging activity, or metal-chelating activity and reducing activity^37^. The highest DPPH radical scavenging activity was observed in cookies containing the highest concentration of APP (C3), while in the control (C0), it was comparatively lower.

#### Reducing power

The values of reducing power of WF, APP, formulations and cookies are given in Table 3. The reducing ability of APP (0.66 mg AAE/g) was significantly (*p* < 0.05) greater than that of WF (0.17 mg AAE/g). Similarly, like other antioxidant assays, the reducing power of formulations exhibited a significant (*p* < 0.05) increase upon the addition of APP to WF.

Baking resulted in a significant (*p* < 0.05) increase in the reducing power of cookies, from 0.41 to 0.51 mg AAE/g. The highest reducing power was found in cookies containing 15% APP. The increase in reducing power with rise in concentration of apple pomace, may be due to enhancement in the level of sugars and polyphenolic compounds, resulting in formation of more melanoidins. Jan et al.^38^ reported an increase in the reducing power of wheat flour cookies blended with buckwheat flour upon baking, which is consistent with the findings of the current study.

#### Lipid peroxidation

Inhibition of lipid peroxidation (ILP) by APP, formulations and cookies is shown in Table 3. APP exhibited the highest ILP value (67%) than the wheat flour (54%). Higher polyphenols in apple pomace might be reason for its enhanced ILP than the WF. The percent ILP of the formulations was observed to increase from 54.0 to 65.0% with the increase in the addition of APP. It has been reported by^39^ that phenolics have the ability to preserve chain-breaking antioxidants and to prevent autocatalytic chain reaction of lipid peroxidation as well which is responsible for improved ILP by the formulations containing APP in our study.

ILP of all cookies decreased significantly (*p* < 0.05) upon baking from 54.00 to 50.00%, 59.35–53.00%, 61.70–55.00% and 65.05–57.00%, respectively for C_0_, C_1_, C_2_ and C_3_. The reduction in ILP of cookies might be ascribed to the increase in lipid oxidation upon baking^40^. The results are in line with those reported by^41^ for whole wheat flour cookies.

#### Metal chelating (Fe2+) activity

The metal chelation activity of APP against Fe^2+^ (1.3%) was slightly higher compared to WF (1.1%) and showed a non-significant (*p* > 0.05) increase from 1.1 to 1.7% with the incorporation of APP from 0 to 10% (Table 3). The presence of some higher potent chelating component(s) in wheat flour like ferulic acid might be responsible for its higher chelating capacity^38^. However, at a concentration of 15% APP, there was a significant (*p* < 0.05) increase in metal chelation activity compared to the control (WF). Zielinska & Turemko^42^. suggested that some phenolic compounds exhibit antioxidant activity by chelating metal ions, potentially explaining the enhanced metal chelation activity observed in formulations with added APP. The phenolic compounds exhibited redox properties (i.e. act as reducing agents, hydrogen donators and singlet oxygen quenchers) and are responsible of metal chelating activity^43^. Furthermore, the synergistic effect which could exist between different antioxidants means that the total antioxidant effect may be greater than the sum of the individual antioxidant activities and the isolation of one compound will not exactly reflect the overall action.

The metal chelation activity of cookies significantly increased (*p* < 0.05) upon baking. This enhancement in metal chelating activity may be attributed to the production of new compounds, such as melanoidins, during thermal processing^36^. Also, as reported by^44^ that baking of chapatti led to a significant increase in metal chelating activity up to 13% with highest increase being observed for chapatti prepared from wheat flour. Cookies containing 15% APP (C3) exhibited the highest metal chelating activity, followed by C2 and C1. This could be due to the higher percentage of apple pomace in C3, which is known to be an excellent source of polyphenols.

### Physicochemical properties of cookies

#### Proximate composition of cookies

The moisture and ash contents of the cookies exhibited significant variation (*p* < 0.05), ranging from 1.4 to 2.8% and 1.49–1.75%, respectively (Table 4). The increased ash content in the cookies may be attributed to the addition of APP to WF. Fibres are known to be rich in minerals, and their incorporation contributes to an increase in the mineral content, thus explaining the rise in ash content observed in cookies containing APP.

**Table 4:** Proximate composition and physical properties of wheat flour cookies supplemented with APP (*n* = 3).

	C_0_	C_1_	C_2_	C_3_
Moisture (%)	1.4 ± 0.57^a^	2.2 ± 0.41^ab^	2.4 ± 0.24^ab^	2.8 ± 0.08^b^
Ash (%)	0.99 ± 0.03^a^	1.49 ± 0.02^b^	1.60 ± 0.16^b^	1.75 ± 0.01^c^
Protein (%)	5.54 ± 0.82^d^	5.25 ± 0.20^c^	4.96 ± 0.08^b^	4.67 ± 0.01^a^
Fat (%)	15.0 ± 0.82^a^	18.0 ± 1.6^b^	19.0 ± 2.5^bc^	20.0 ± 2.7^c^
Fibre (%)	8.00 ± 0.82^a^	16.00 ± 1.6^b^	18.00 ± 2^bc^	20.00 ± 2.4^c^
Weight (g)	11.16 ± 0.01^a^	11.32 ± 0.02^b^	11.53 ± 0.03^c^	11.61 ± 0.04^d^
Diameter (cm)	5.7 ± 0.08^a^	5.8 ± 0.16^a^	5.9 ± 0.24^a^	6.0 ± 0.33^a^
Thickness (cm)	0.6 ± 0.41^a^	0.6 ± 0.33^a^	0.6 ± 0.14^a^	0.6 ± 0.16^a^
Spread ratio	9.50 ± 0.24^a^	9.66 ± 0.02^ab^	9.83 ± 0.01^bc^	10.0 ± 0.08^c^
Texture (kg)	5.67 ± 0.04^d^	4.94 ± 0.03^c^	4.12 ± 0.02^b^	3.88 ± 0.01^a^
Color				
L*	41.38 ± 0.60^d^	39.35 ± 0.76^c^	35.33 ± 2.49^b^	32.17 ± 0.48^a^
a*	7.52 ± 0.56^a^	8.77 ± 0.31^b^	8.68 ± 0.45^b^	9.64 ± 0.18^c^
b*	38.58 ± 0.68^a^	38.60 ± 0.40^a^	39.12 ± 0.50^b^	39.36 ± 0.40^c^
ΔE	–	2.38 ± 0.03^c^	6.18 ± 0.05^b^	9.48 ± 0.34^a^

Results are expressed as means (*n* = 3) ± standard deviation.

*C*_*0*,_*C*_*1*,_*C*_*2*_* and C*_*3*_ wheat flour cookies containing 0%, 5%, 10% and 15% APP respectively.

Values followed by same letter in a row do not differ significantly (*p* < 0.05).

The fat and protein contents of the cookies containing APP showed non-significant variation (*p* > 0.05) compared to C0, ranging from 15 to 20% for fat and 4.67–5.54% for protein, respectively. Bhat et al.^41^ reported a protein content of 6.13% in whole wheat flour cookies, slightly higher than the observation in our study. Furthermore, the fibre content of cookies significantly increased (*p* < 0.05) from 8 to 20% with the concentration of APP ranging from 0 to 15%. Rupasinghe et al.^45^ found that the addition of apple skin powder enhanced the fibre content of muffins, corroborating the findings of the current study.

#### Physical properties of cookies

Weight, thickness, diameter and spread ratio of the cookies are given in Table 4. The weight of the cookies increased significantly (*p* < 0.05) from 11.16 g to 11.61 g with the addition of APP in all formulations. This increase could be attributed to the enhanced water holding capacity of APP, given its rich fibre content. Pinki & Awasthi^46^, reported a similar increase in the weight of cakes after the addition of beetroot pomace, supporting our findings.

Moreover, the spread ratio and diameter of the cookies exhibited significant (*p* < 0.05) variation, increasing from 9.50 to 10 and 5.7 to 6.0 cm, respectively, with the addition of APP. These findings are consistent with studies conducted by^*19*^ and^*21*^, who reported similar results for cookies blended with sesame peel and carrot pomace powder, respectively. The results suggest that the incorporation of APP in cookie formulations resulted in significant changes in weight, thickness, diameter, and spread ratio, highlighting its impact on the physical characteristics of the cookies.

#### Texture

Table 4 presents the hardness of freshly prepared cookies supplemented with APP, which decreased significantly (*p* < 0.05) from 5.67 kg to 3.88 kg compared to the control. This reduction can be attributed to the high fibre content in apple pomace, which possesses greater water holding capacity than WF. Consequently, this diminishes the amount of water available for gluten development, leading to the formation of a weaker gluten network.

Van Der Sman & Broeze^47^, noted that a higher amount of soluble fibre in apple pomace acts as a lubricant, decreasing the hardness of rice crackers and resulting in a crispier texture rather than a harder one. These findings are consistent with those reported by^48^ in puffed snacks containing apple pomace.

#### Color

The Hunter color L*, a*, and b* values of the control cookies (C0) were measured as 41.38, 7.52, and 38.58, respectively (Table 4). The L* value of the cookies significantly decreased (*p* < 0.05) from 41.38 to 32.17 with the increasing addition of APP from 0 to 15% compared to the control. Conversely, the a* value of the cookies significantly increased (*p* < 0.05) from 7.52 to 9.64, while the b* value showed a non-significant increase from 38.58 to 39.36 with the addition of APP. The increased redness (a*) of the cookies may be attributed to the oxidation of polyphenols present in apple pomace, as polyphenols serve as a substrate for enzymatic browning. Enzymatic browning is more responsible for the development of redness and yellowness in the samples than non-enzymatic browning^49^. These findings align with the results reported by^4^ for biscuits containing mango peel powder.

The total color difference (∆E) of cookies increased with an increasing proportion of apple pomace (2.38–9.48). Because of the natural dark color of apple pomace and its increasing amount, it was evident that the pomace greatly altered the color of the cookies. It is clear from the table that on increasing the apple pomace concentration the difference in color with respect to control (C_0_) increased drastically. Similar results were obtained in the study by^50^ who analysed the effect of the addition of apple pomace on the properties of crackers.

### Sensory evaluation

The sensory properties of cookies added with APP were assessed on the basis of appearance, texture, mouthfeel, flavor and overall acceptability (Table 5). It was observed that in comparison to C_1,_ C_2_ and C_3_ cookies, C_0_ cookies were ranked lowest by sensory panellists for all sensory attributes. Cookies with 10% APP (C_2_) gained the maximum score for all sensory characteristics. In a study carried out by^51^ the sensory scores for flavor enhanced with increase in apple pomace concentration which is similar to our findings. The results of overall sensory properties showed that the cookies with added APP can be prepared with enhanced nutritional value and health benefits for consumers, without compromising the organoleptic characteristics.

**Table 5:** Sensory characteristics of wheat flour cookies supplemented with APP.

	C_0_	C_1_	C_2_	C_3_
Appearance	8.02 ± 0.01^c^	7.99 ± 0.02^b^	7.72 ± 0.03^b^	7.15 ± 0.04^a^
Mouth feel	7.21 ± 0.82^a^	8.26 ± 0.08^b^	9.12 ± 0.02^c^	9.03 ± 0.01^c^
Flavor	7.09 ± 0.82^a^	8.12 ± 0.02^b^	8.15 ± 0.03^b^	8.33 ± 0.05^c^
Texture	7.04 ± 0.08^a^	7.07 ± 0.49^a^	8.12 ± 0.02^b^	7.68 ± 0.01^c^
Overall acceptability	7.31 ± 0.82^a^	7.85 ± 0.08^ab^	8.27 ± 0.04^c^	8.04 ± 0.01^bc^

Results are expressed as means (*n* = 3) ± standard deviation.

*APP* apple pomace powder; *C*_*0*,_*C*_*1*,_*C*_*2*_* and C*_*3*_ wheat flour cookies containing 0%, 5%, 10% and 15% APP respectively.

Values followed by same letter in a row do not differ significantly (*p* < 0.05).

## Conclusion

Incorporating apple pomace powder (APP) into wheat flour significantly altered its physicochemical, functional, and antioxidant attributes. Notably, the water absorption capacity (WAC) and swelling power (SP) of the wheat flour increased significantly post-APP addition, alongside rises in onset, peak, and endset temperatures. Rheological analysis unveiled frequency-dependent behavior in the wheat flour dough.

The enriched cookies exhibited heightened antioxidant properties and total phenolic content while maintaining sensory excellence akin to the control. Notably, cookies with 10% APP garnered the highest scores across all sensory attributes on the 9-point hedonic scale, indicating superior sensory appeal. Overall, the study underscores the potential for producing health-enhancing cookies by utilizing fruit and vegetable waste from various food industries globally. These waste products serve as abundant sources of bioactive compounds, augmenting consumer health benefits without compromising product organoleptic qualities. Moreover, their utilization contributes to the reduction of environmental pollution through the repurposing of underutilized food waste by-products.

## Data Availability

All data generated or analysed during this study are included in this published article.
